# Supplementary material for: Meta-analysis of the correlation between serum uric acid level and carotid intima-media thickness
Source: PLoS One. 2021 Feb 11;16(2):e0246416. doi: 10.1371/journal.pone.0246416 (PMC7877574; doi:10.1371/journal.pone.0246416)
Supplement: S1 Appendix — (DOCX) [file pone.0246416.s001.docx]

**S2 Appendix. Subgroup analysis of forest plots based on TC、SBP、DBP、Triglyceride and LDL-C.**


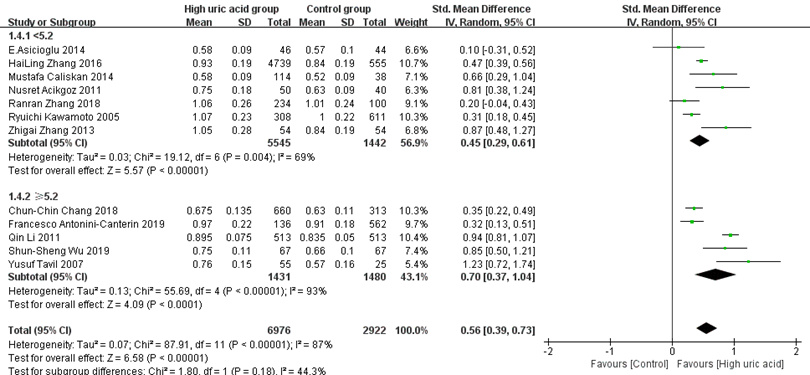


1. TC


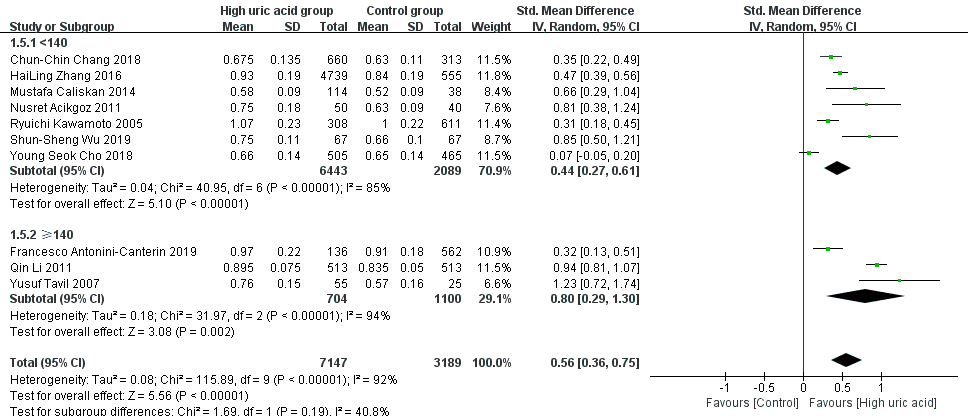


1. SBP


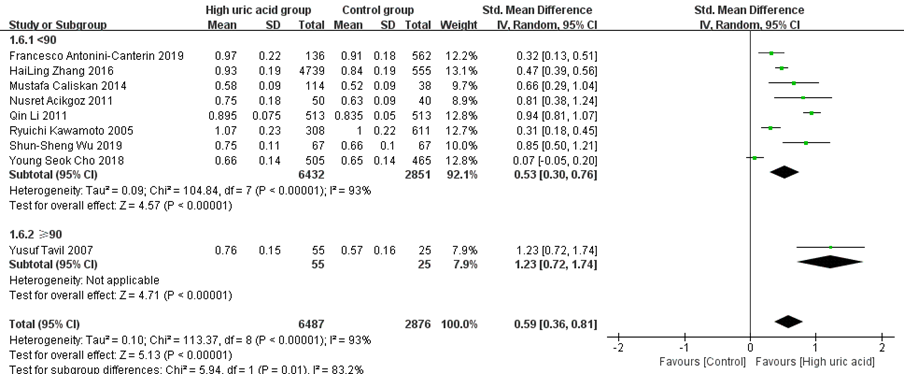


1. DBP


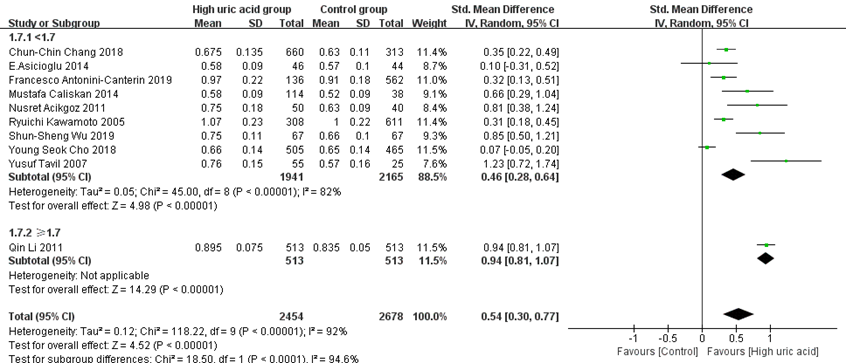


1. Triglyceride


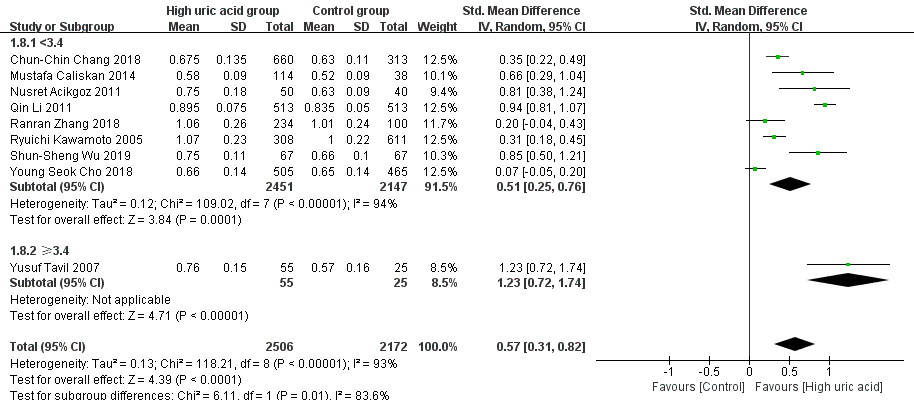


1. LDL-C
