# Supplementary material for: Meta-analysis of the correlation between serum uric acid level and carotid intima-media thickness
Source: PLoS One. 2021 Feb 11;16(2):e0246416. doi: 10.1371/journal.pone.0246416 (PMC7877574; doi:10.1371/journal.pone.0246416)
Supplement: S1 Table — (DOCX) [file pone.0246416.s003.docx]

**S1 Table. Summary of the included literature characteristics and key findings.**

| **N** | **Author** | **Journal** | **Type** | **Publication date** | **Age** | **Male(%)** | **Main findings** |
| --- | --- | --- | --- | --- | --- | --- | --- |
| 1 | Ranran Zhang[20] | Qingdao university | cross-sectional | 2018.03.09 | 59.5±7.9 | 57.18% | **1.**With the increase of SUA level, the probability of carotid plaque was increased (OR=1.003, P<0.05) |
| 2 | Hailing Zhang[21] | Fujian Yike Daxue Xuebao | cross-sectional | 2017.08.28 | 55±11.59 | 60.01% | **1.**In women, uric acid levels are associated with thickening of cIMT, and hyperuricemia may make atherosclerosis more likely |
| 3 | Zhigai Zhang[22] | Zhonghua Shiyong Yiyao Zazhi | cross-sectional | 2013.02.28 | 52.5±12(40-77) | 56.48% | **1.**Blood uric acid can cause the carotid intima lesion in type 2 diabetes mellitus, and is the main factor |
| 4 | Chunyu Yang[23] | Hebei Yixue | cross-sectional | 2005.06.30 | -- | -- | **1.**Compared with the control group, the carotid intima thickness in the group with higher uric acid level increased, and the incidence of carotid plaque increased, but there was no significant difference |
| 5 | Francesco Antonini-Canterin[24] | Monaldi Archives for Chest Disease | cross-sectional | 2019.08.04 | 57.3±13.7 | 45.10% | **1.**SUA levels correlated with IMT (r=0.225,P<0.001); **2.**the correlations were statistically significant in males and females |
| 6 | Qin Li[25] | Cardiovascular Diabetology | cross-sectional | 2011 | 65.6±11.7(41-92) | 39.12% | **1.**Serum uric acid level was negatively correlated with carotid intima al thickness (P=0.02);  **2.**Serum uric acid level is an independent risk factor for carotid atherosclerosis in type 2 diabetes mellitus |
| 7 | Chun-Chin Chang[26] | Scientific Reports | longitudinal | 2018.08.27 | 61.7±8.6(50-89) | 46.80% | **1.**The group with higher serum uric acid levels had a higher carotid intima thickness and an increased risk of cardiovascular disease |
| 8 | Kumral.E[27] | Acta Neurologica Scandinavica | cross-sectional | 2013.11.07 | 66±11(40-90) | 60% | **1.**Compared with people with normal uric acid level, patients with hyperuricemia have a higher incidence of carotid artery disease (OR=1.8, 95%CI:[1.1 -- 3.1]; P = 0.01); **2.** The mean intima thickness of carotid artery was significantly thickened |
| 9 | Young Seok Cho[28] | CrossMark | cross-sectional | 2018.08.28 | 52.7±6.8 | 94.70% | **1.**Elevated serum UA in asymptomatic adults is associated with increased carotid FDG uptake, which suggests a potential role of UA in carotid inflammatory atherosclerosis. |
| 10 | Ryuichi Kawamoto[29] | Internal Medicine | longitudinal | 2005.04.21 | 74.3±8 | 43.30% | **1.**Serum uric acid is a risk factor or marker for ultrasonographically determined IMT |
| 11 | Mustafa Caliskan[30] | Acta Cardiologica | cross-sectional | 2014 | 44.6±7.9 | 85.40% | **1.**Elevated uric acid levels were associated with increased carotid IMT;  **2.**Elevated serum uric acid levels might contribute to the increase in cardiovascular risk in masked hypertension |
| 12 | E.Asicioglu[31] | Transplantation Proceedings | cross-sectional | 2014 | 41.5±11 | -- | **1.**Compared with the control group, the recipients of kidney transplantation had higher serum uric acid level but no significant difference in carotid intimal thickness |
| 13 | Nusret Acikgoz[32] | Medical Principles and Practice | cross-sectional | 2011.11.09 | 51.8±3.2 | 35% | **1.**Serum uric acid levels were higher in patients with CSX and elevated serum uric acid levels were associated with carotid atherosclerosis |
| 14 | Yusuf Tavil[33] | Atherosclerosis | cross-sectional | 2007.04.09 | 50.3±11 | 56.25% | **1.**Higher SUA levels are associated with atherogenesis independent from hypertension |
| 15 | Shun-Sheng Wu[34] | Oxidative Medicine and Cellular Longevity | cross-sectional | 2019.10.09 | 50.8±4.3 | 51.49% | **1.**There was a positive correlation between serum uric acid level and carotid intimal thickness (R =0.382,P<0.001) |
