# Supplementary material for: Meta-analysis of the correlation between serum uric acid level and carotid intima-media thickness
Source: PLoS One. 2021 Feb 11;16(2):e0246416. doi: 10.1371/journal.pone.0246416 (PMC7877574; doi:10.1371/journal.pone.0246416)
Supplement: S2 Table — (DOCX) [file pone.0246416.s004.docx]

| N | Author | Confounding factors |
| --- | --- | --- |
| 1 | Ranran Zhang[23] | FPG, TC, TG, HDL-C, LDL-C |
| 2 | Hailing Zhang[22] | Height, Weight, SBP, DBP, FPG, TC, TG, HDL-C, LDL-C, Cr, Smoking, Drinking, Salt, Exercise situation |
| 3 | Zhigai Zhang[24] | FPG, TC, TG, HDL-C, LDL-C, UAER |
| 4 | Chunyu Yang[21] | -- |
| 5 | Francesco Antonini-Canterin[26] | SBP, DBP, TC, HDL, LDL, Triglyceride, Index of stiffness Beta, Glucose, Active Smoking, Hypertension, Dyslipidemia, Diabetes mellitus, Obesity |
| 6 | Qin Li[31] | Smoking, Alcohol, Duration of diabetes, Family history of diabetes, SBP, DBP, BMI, Waist circumference, Waist to hip ratio, Glucose, Insulin, HOMA-IR , CRP, Cr, Estimated GFR, ACR, Triglyceride, TC, HDL-C, LDL-C |
| 7 | Chun-Chin Chang[20] | HDL-C, LDL-C, TC, SBP, Smoking, BMI, Triglyceride, HbA1c, hs-CRP |
| 8 | Kumral.E[30] | Retinal ischemia, Lower HDL-C, Higher LDL-C, Cause of stroke, Hypertension, Diabetes mellitus, smoking, History of CHD, Atrial fibrillation, Hypercholesterolemia, Hypertriglyceridemia, Lower apo A, Higher apo B, Lipoprotein (a), Hyperhomocysteinemia, Obesity, Diabetic foot, Physical activity absence, Renal failure |
| 9 | Young Seok Cho[28] | Hypertension, Diabetes, Smoker, BMI, Waist circumference, SBP, DBP, Estimated GFR, Cholesterol, Triglyceride, HDL-C, LDL-C, hsCRP |
| 10 | Ryuichi Kawamoto[29] | BMI, Obesity, Smoking, SBP, DBP, Antihypertensive drug use, Hypertension, Triglyceride, TC, Antilipidemic drug use, HDL-C, LDL-C, Dislipidemia, Diabetes mellitus, Atherosclerotic disease(Ischemic stroke, Ischemic heart disease) |
| 11 | Mustafa Caliskan[27] | BMI, Heart rate, Office SBP, Office DBP, Ambulatory 24-h SBP, Ambulatory 24-h DBP, Ambulatory daytime SBP, Ambulatory daytime DBP, Ambulatory nighttime DBP, TC, HDL-C, LDL-C, Triglyceride, hs-CRP, Cr, Fasting blood glucose |
| 12 | E.Asicioglu[34] | BMI, Glucose, Cr, MDRD, TC, Triglyceride, HDL, LDL, WBC, Hemoglobin, Ferritin, CRP, Albumin, Ca, P, ALP, PTH, 25-OH-D, FGF-23, |
| 13 | Nusret Acikgoz[25] | BMI, Smoking, Systolic BP, Diastolic BP, Glucose, TC, HDL-C, LDL-C, Triglyceride, Cr |
| 14 | Yusuf Tavil[32] | BMI, Heart rate, Smoking, TC, HDL-C, LDL-C, triglyceride, SBP, DBP, Plasma creatinine, Fasting glucose, Cardiovascular medication(ASA, Beta-blockers, ACE inhibitors or Angiotensin receptor blocker, Ca^2+^ -antagonists, Cholesterol-lowering drugs) |
| 15 | Shun-Sheng Wu[33] | Smoking, Alcohol, BMI, Waist, SBP, DBP, MDA, SOD, 8-OHdG, hs-CRP, Fasting blood sugar, Hemoglobin A1c, Triglyceride, TC, HDL-C, LDL-C, Tchol_HDL ratio, WBC, AST, ALT, Renal GFR, Cr |

**S2 Table. Confounding factors in the 15 references included in the meta-analysis.**

Abbreviations: FPG, fasting plasma glucose; TG, triacylglycerol; TC, total cholesterol; HDL-C, high-density lipoprotein cholesterol; LDL-C, low-density lipoprotein cholesterol; LDL,

Low-density lipoprotein; HDL, high-density lipoprotein; Cr, creatinine; DBP, diastolic blood pressure; SBP, systolic blood pressure; UAER, urinary albumin ejection rate; HbA1c, glycated haemoglobin; MDRD, modification of diet in renal disease equation; WBC, white blood cells; Ca, calcium; P, phosphorus; ALP, alkaline phosphatase; CHD, coronary heart disease; hs-CRP, high-sensitivity C-reactive protein; ACR, albumin/creatinine ratio; MDA, malondialdehyde; SOD, superoxide dismutase; 8-OHdG, 8-hydroxy-2′-deoxyguanosine; GFR, glomerular filtration rate; hsCRP, high-sensitivity C-reactive protein; ACE, angiotensin-converting enzyme; ASA, acetylsalicylic acid
