# Supplementary material for: Meta-analysis of the correlation between serum uric acid level and carotid intima-media thickness
Source: PLoS One. 2021 Feb 11;16(2):e0246416. doi: 10.1371/journal.pone.0246416 (PMC7877574; doi:10.1371/journal.pone.0246416)
Supplement: Attachment — Submitted filename: Response to Reviewers.docx [file pone.0246416.s006.docx]

**Editor:**

***Any new text in the revised manuscript has been noted below in bold font.**

The Discussion section has been modified according to the suggestions of reviewers.

**RESPONSE TO REVIEWER’S COMMENTS:**

**Reviewer #3:** The discussion could be more concise and highlight its clinical significance, in addition, there is a little no more revision needed.

**Reply:** We appreciate the excellent observation from Reviewer #3 regarding the Discussion section. We adopted the suggestions of reviewers, revised the discussion and added the following contents to highlight the clinical significance (Discussion, lines 244-251):

“Therefore, this study revealed that a high level of SUA may be a predictor of atherosclerosis, and strengthening the control of serum uric acid levels in early prevention is helpful to reduce the risk of atherosclerosis. These findings proved that the rising level of SUA could pose an increased risk of cardiovascular and cerebrovascular diseases, provided a potential therapeutic target and new ideas for further clinical researches. It was suggested that decreasing level of uric acid could be an adjunctive therapy for cardiovascular and cerebrovascular diseases, and the atherosclerosis diagnosis and treatment also could be supported by testing the level of SUA in clinic.”
